# Supplementary material for: Inflammatory factors and risk of lung adenocarcinoma: a Mendelian randomization study mediated by blood metabolites
Source: Front Endocrinol (Lausanne). 2024 Aug 27;15:1446863. doi: 10.3389/fendo.2024.1446863 (PMC11384989; doi:10.3389/fendo.2024.1446863)
Supplement: Supplementary file 1 [file DataSheet1.zip › supplementary files/S2/GCST90200324/forest.pdf]

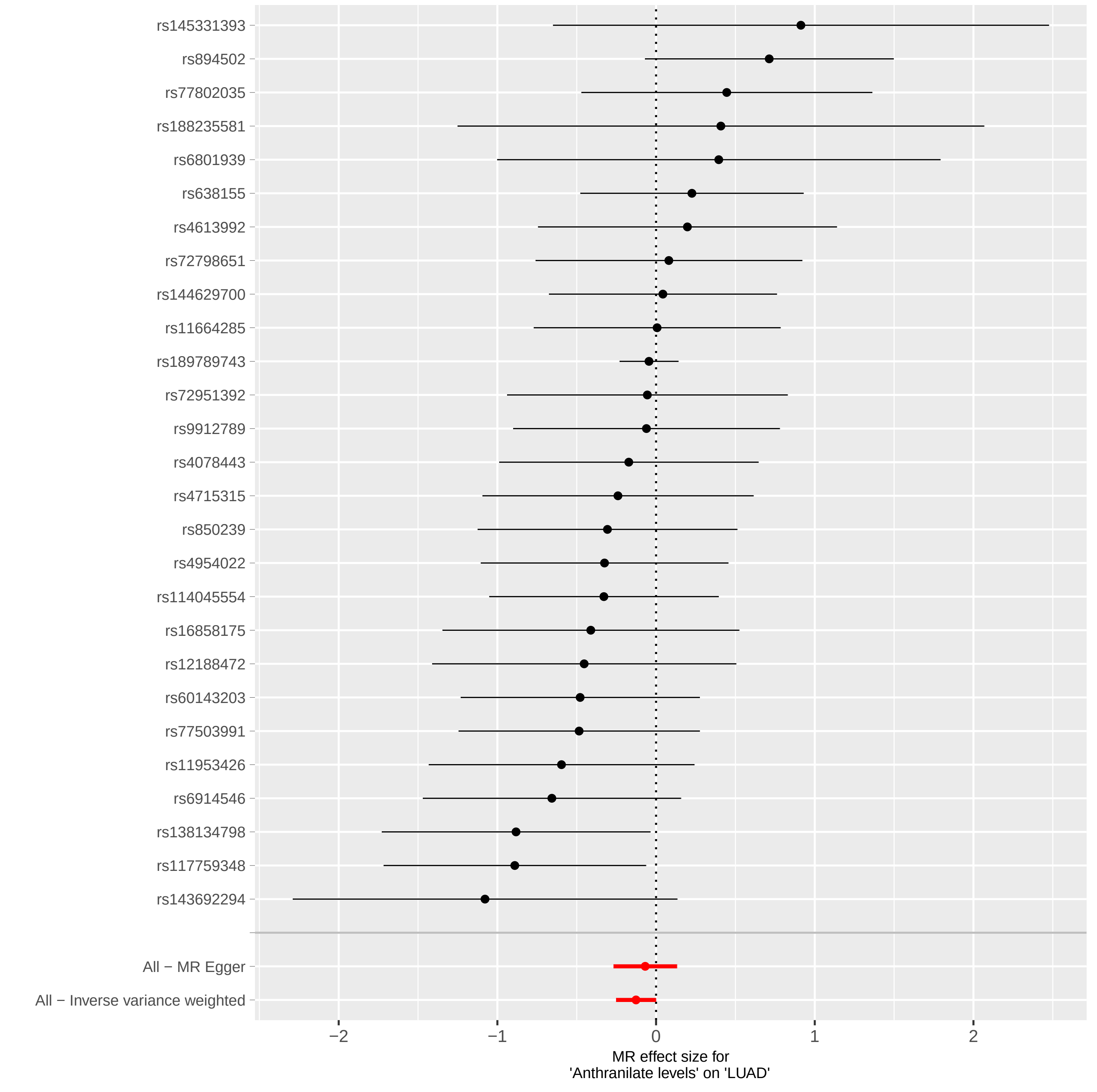
rs145331393
rs894502
rs77802035
rs188235581
rs6801939
rs638155
rs4613992
rs72798651
rs144629700
rs11664285
rs189789743
rs72951392
rs9912789
rs4078443
rs4715315
rs850239
rs4954022
rs114045554
rs16858175
rs12188472
rs60143203
rs77503991
rs11953426
rs6914546
rs138134798
rs117759348
rs143692294
All − MR Egger
All − Inverse variance weighted
−2
−1
0
1
2
MR effect size for
'Anthranilate levels' on 'LUAD'
